# Agricultural activities and risk of Alzheimer’s disease: the TRACTOR project, a nationwide retrospective cohort study

**DOI:** 10.1007/s10654-023-01079-0

**Published:** 2024-01-10

**Authors:** Pascal Petit, Elise Gondard, Gérald Gandon, Olivier Moreaud, Mathilde Sauvée, Vincent Bonneterre

**Affiliations:** 1 CNRS, UMR 5525, VetAgro Sup, Grenoble INP, CHU Grenoble Alpes, TIMC, Univ. Grenoble Alpes, 38000 Grenoble, France; 2 Present Address: Centre Régional de Pathologies Professionnelles et Environnementales, CHU Grenoble Alpes, 38000 Grenoble, France; 3 Present Address: AGEIS, Univ. Grenoble Alpes, 38000 Grenoble, France; 4 Centre Mémoire de Ressources et de Recherche, CHU Grenoble Alpes, 38000 Grenoble, France; 5 Laboratoire de Psychologie et Neurocognition, UMR 5105, CNRS, LPNC, Univ. Grenoble Alpes, Univ. Savoie Mont Blanc, 38000 Grenoble, France

**Keywords:** Administrative health database, Digital electronic health/medical record, Insurance claims, Agriculture, Farming, Alzheimer’s disease, Dementia, Health surveillance, Occupational activity, Big data, Data mining, Cohort, Epidemiology, Population-based, Nationwide, France, Europe

## Abstract

Data regarding Alzheimer’s disease (AD) occurrence in farming populations is lacking. This study aimed to investigate whether, among the entire French farm manager (FM) workforce, certain agricultural activities are more strongly associated with AD than others, using nationwide data from the TRACTOR (Tracking and monitoring occupational risks in agriculture) project. Administrative health insurance data (digital electronic health/medical records and insurance claims) for the entire French agricultural workforce, over the period 2002–2016, on the entire mainland France were used to estimate the risk of AD for 26 agricultural activities with Cox proportional hazards model. For each analysis (one for each activity), the exposed group included all FMs that performed the activity of interest (e.g. crop farming), while the reference group included all FMs who did not carry out the activity of interest (e.g. FMs that never farmed crops between 2002 and 2016). There were 5067 cases among 1,036,069 FMs who worked at least one year between 2002 and 2016. Analyses showed higher risks of AD for crop farming (hazard ratio (HR) = 3.72 [3.47–3.98]), viticulture (HR = 1.29 [1.18–1.42]), and fruit arboriculture (HR = 1.36 [1.15–1.62]). By contrast, lower risks of AD were found for several animal farming types, in particular for poultry and rabbit farming (HR = 0.29 [0.20–0.44]), ovine and caprine farming (HR = 0.50 [0.41–0.61]), mixed dairy and cow farming (HR = 0.46 [0.37–0.57]), dairy farming (HR = 0.67 [0.61–0.73]), and pig farming (HR = 0.30 [0.18–0.52]). This study shed some light on the association between a wide range of agricultural activities and AD in the entire French FMs population.

**Supplementary Information:**

The online version contains supplementary material available at 10.1007/s10654-023-01079-0.

## Introduction

Alzheimer’s disease (AD) is the most common type of dementia, which affects millions of adults worldwide and causes progressive and irreversible brain damage [1–4]. AD is a complex, multifactorial, and heterogeneous disorder that involves many risk factors. The interaction of genetic susceptibility and environmental factors throughout the entire life course (exposome) contributes to the onset and/or development of AD [4–6]. Several established and possible risk factors have been identified. Aging, sex, genetic predisposition, and ancestry are recognized risk factors that are non-modifiable [1, 5, 7, 8]. Many potentially modifiable risk factors have been identified. Some of these possible risk factors are linked to lifestyles (e.g. smoking, physical inactivity, social isolation, low education status, and/or low occupational complexity), to the diet (e.g. gut microbiome, coffee, and alcohol consumption), and to cardiovascular risk factors (e.g. diabetes, midlife hypertension, and obesity) [1–3, 5, 7, 9–12]. Exposure to chemicals, such as air pollution, metals, pesticides, and nanoparticles, are possible risk factors suspected to be directly or indirectly associated with the pathogenesis of AD [4, 5, 10, 13–24].

The association between AD and environmental factors, in particular pesticides, has drawn considerable attention this last decade [16, 23, 25–27]. However, contrary to Parkinson’s disease (PD) [13, 28, 29], evidence regarding the role of pesticides on the onset and development of AD is limited and more inconsistent. Some pesticides have been associated with higher risks of AD, in particular DDT [1], carbamates, organophosphates (OPs), and organochlorines [10]. Many pesticides are neurotoxic in nature and share common characteristics, such as the ability to induce mitochondrial dysfunction, oxidative stress, and neuronal loss, as well as α-synuclein aggregation and fibrillation [21, 23, 30–32]. In addition, some pesticides, in particular OPs, inhibit acetylcholinesterase, which results in various toxic effects on the central nervous system [10, 12–16, 29, 33, 34]. In 2021, a French collective expert review concluded there was a moderate link between occupational exposure to pesticides and AD [34]. To the best of our knowledge, no other expert reviews have come to similar conclusions. Therefore, there is a need to expand the research on identifying environmental risk factors, in particular in farming populations, where occupational exposures and some of the aforementioned possible risk factors could play an important role in the onset and/or development of AD. Besides pesticides, other possible risk factors for AD and other related dementias (ADRD) prominent in agriculture exist, in particular exposure to metals, hearing loss, and psychosocial factors such as depression and social isolation [2, 6, 7, 25, 35]. Indeed, farmers can be exposed to excessive noise from grain dryers or tractors, and several studies have shown that farmers have a higher prevalence of depression when compared to non-farmers [35, 37]. However, studies investigating AD in agriculture are limited and pertain only to a small proportion of the agricultural workforce [37–40].

The goal of this study was to investigate whether, among the entire French farm manager (FM) population, certain agricultural activities are more strongly associated with AD than other activities, overall and by sex category, on the whole French metropolitan territory.

## Methods

### Data source

We used data from an administrative health database from the Mutualité Sociale Agricole (MSA) available to the TRACTOR project, which pertained to the entire French agricultural workforce, over the period 2002–2016, on the entire mainland of France [41]. In France, the National Health Insurance Fund for Agricultural Workers and Farmers (MSA) pertains to 5% of the overall population and covers the entire agricultural workforce [42]. This health insurance is compulsory for all agricultural workers and ensures full coverage of all medical expenses and most health care expenditures. Briefly, insurance data on contributors demographic characteristics and health were routinely collected by MSA and made available to TRACTOR. Occupational activities were coded according to an internal MSA coding system referring to 26 different activities (e.g. pig farming) (Table S1) [43]. Health data (electronic health/medical records) pertained to declared chronic diseases/long-term illnesses (LTI), such as AD, for which FMs are entitled to fee exemption and full coverage of health care expenditures. When an individual suffers from a LTI from a list of 30 illnesses [44], this person can be eligible for LTI attribution. AD is one of the eligible diseases under the LTI scheme. Once the diagnosis of AD has been established by a neurologist, a detailed request can be addressed to the medical department of the health insurance system by the GP to obtain the LTI status for the patient. For the LTI to be granted, the AD diagnosis must be validated by the insurance physician who is using stringent criteria, either on the basis of clinical and additional medical examinations reported by the neurologist, or on the original medical records if any doubt exists [44–46]. For AD, the LTI status is granted for 10 years and is renewable. If the LTI is granted, the LTI is assigned an ICD-10 code (10th revision of the International Statistical Classification of Diseases and Related Health Problems), and all AD-related care (e.g. drugs, medical exams) is 100% reimbursed. For individuals suffering from AD, the LTI request is not always made. For individuals without LTI, all AD-related care is not 100% reimbursed. Health data pertaining to reimbursed drugs (with or without LTI), medical prescriptions, and medical exams, with the exception of those dispensed by or at the hospital, were also available to the TRACTOR project. The data were analyzed from January 2021 to October 2022. This study was approved by the French independent administrative authority protecting privacy and personal data, and all methods were performed in accordance with the relevant guidelines and regulations. As MSA provided data after encryption to protect private information, the need for informed consent was waived. This study followed the Strengthening the Reporting of Observational Studies in Epidemiology (STROBE) reporting guideline (supplementary information).

### Study population and outcome

FM is a generic term that includes farm or company managers, owners, and self-employed persons. A FM is a person who owns and/or oversees a farm while working in the field on various activities such as operating a tractor, picking vegetables, or applying pesticides. Most FMs are participating in daily tasks in the field, but their degree of involvement varies from one individual to another, depending on several factors, such as the type of activity (e.g. dairy farming), the number of employees, or the farm surface. This degree of involvement is not recorded by MSA. Each year, FMs are asked by MSA to declare their main annual activity (in terms of effective working time) based on an internal labor coding system used by MSA consisting of 26 possible choices (Table S1) [43]. To the best of our knowledge, there are no incentives for FMs to report their main activity one way or another. All FMs who performed at least one of these 26 activities once (1 yearly declaration to MSA) between 2002 and 2016, were included. The agricultural activity was considered a proxy for occupational exposure. For each activity, the duration of exposure was defined as the number of years (number of yearly declarations to MSA) an individual performed this given activity between 2002 and 2016.

AD cases were identified either using ICD-10 codes for FMs declared with AD through the LTI insurance declaration scheme or with ATC codes (Anatomical Therapeutic Chemical classification system) referring to AD drugs prescribed to FMs (with or without LTI). FMs were considered to have AD if they had at least one LTI declaration for AD or at least one prescription of any drugs used to treat AD (Table 1). Health data (LTI declarations and drug prescriptions) from 2012 to 2016 were used as a follow-up period, with January 1st, 2012, as the baseline time point (i.e. time zero) and December 31st, 2016, as the follow-up end. The number of prevalent cases was defined as the number of AD cases at time 0, while incident AD cases were defined as cases occurring after time 0.

**Table 1:** ICD-10 and ATC codes used for identifying AD cases among farm managers

Data origin	Classification system	Code	Definition	Drug indication	Analysis
Long-term illness scheme/declaration (requirement to be considered as an AD case: at least one declaration of any of these three ICD-10 codes)	ICD-10	G30	Alzheimer’s disease	–	MA, SA1 to SA6
	ICD-10	F00	Dementia in Alzheimer’s disease	–	MA, SA1 to SA6
	ICD-10	F01	Vascular dementia	–	SA3
	ICD-10	F02	Dementia in other diseases classified elsewhere	–	SA3
	ICD-10	F03	Unspecified dementia	–	MA, SA1 to SA4, and SA6
Drug prescription for AD (requirement to be considered as an AD case: at least one prescription of any of these five ATC codes)	ATC	N06DA02	Donepezil	For AD only	MA, SA1 to SA5, SA7
	ATC	N06DA03	Rivastigmine	For AD and PD	MA, SA1 to SA5, SA7
	ATC	N06DA04	Galantamine	For AD only	MA, SA1 to SA5, SA7
	ATC	N06DA52	Donepezil and memantine	For AD only	MA, SA1 to SA5, SA7
	ATC	N06DA53	Donepezil, memantine and ginkgo folium	For AD only	MA, SA1 to SA5, SA7
	ATC	N06DX01	Memantine	For AD only	MA, SA1 to SA5, SA7

AD, Alzheimer’s disease; ATC, anatomical therapeutic chemical classification system; ICD-10, 10th revision of the International Statistical Classification of Diseases and Related Health Problems; MA, main analysis; PD, Parkinson’s disease; SA, sensitivity analysis

### Statistical analysis

To determine whether certain agricultural activities are more strongly associated with AD than other activities, Cox proportional hazards model was used, with time to the first AD insurance declaration or AD drug prescription as the underlying timescale. AD risks were estimated, with hazard ratios (HRs) and 95% confidence intervals, according to activities when the number of exposed cases was ≥ 10. One model was created for each activity. The dependent variables of the model were the timescale (continuous) and the AD diagnosis (two categories: yes or no). The reference/control group included all FMs who did not carry out the activity of interest, while the exposed group included all FMs that performed the activity of interest. For instance, for crop farming, the reference group included all FMs that never farmed crops between 2002 and 2016, while the exposed group included FMs that were crop farmers at least once from 2002 to 2016. For pig farming, the reference group included all FMs that were never pig farmers between 2002 and 2016, while the exposed group included FMs that were dairy farmers at least once from 2002 to 2016. Immortal time occurs for FMs who perform a main activity for the first time after the beginning of follow-up. To account for this bias, the activity was parameterized as a time-dependent variable in the models (using the counting-process data format approach). The assumption of proportional hazard rate was checked for each model by verifying the independence of scaled Schoenfeld’s residuals and time. When the assumption was not satisfied for a covariate (e.g. age), a covariate*time interaction was added to the model. The median follow-up was estimated using the Kaplan–Meier reverse approach. The Benjamini–Hochberg approach was used to account for multiple testing.

Individual-level covariates recorded in the database were used (Table S2), including age, sex, farm characteristics, and preexisting medical comorbidities, which were based on ICD-10 codes. All analyses were adjusted for age (continuous) and sex (categorical: female and male). All other available covariates were considered for each model based on the variance inflation factor (VIF), with the exclusion of collinear covariates with a VIF > 2.5 (Table S2). One-hot encoding was applied to all categorical variables with more than two categories, with the elimination of one category for each factor to avoid multicollinearity. For all dummy variables (e.g. activity), the no (or 0) was used as reference. Sex-specific analyses (subgroup analyses), with one separate model for each sex, were also conducted to determine sex-specific AD risk estimates.

Seven sensitivity analyses were conducted to test hypotheses and address potential sources of bias. To better assess potential differences due to covariates or differences in exposure, a sensitivity analysis including a common set of covariates (age, sex, and the first year of the farm’s establishment) in all models was performed (SA1). Because AD and other related dementias are not common before age 60, a sensitivity analysis (SA2) included only FMs at risk of AD, that is, individuals who were 60 years and older. Because it is very difficult based on symptoms alone to accurately identify AD, a sensitivity analysis (SA3) including FMs diagnosed with AD and other related dementias was conducted (Table 1). To address potential case misclassification, four other sensitivity analyses were conducted. For SA4, individuals who were prescribed an AD drug (Table 1) that also had a LTI declaration for PD (ICD-10 codes G20 or G21) were not considered as AD cases. For SA5, FMs who were prescribed with an AD drug (Table 1) and who also had a LTI declaration for PD (ICD-10 codes G20 or G21) or a LTI for unspecified dementia (ICD-10 code F03) were not considered as AD cases. For SA6, the AD case identification was restricted to LTI declarations, while for SA7, it was restricted to AD drug prescriptions only (Table 1).

There was no missing data. All statistical analyses were performed with R software 4.1.2® (R Core Team, Vienna, Austria) for Windows 10©.

## Results

### Population characteristics

Baseline characteristics of the study population are presented in Table 2, which provides, by health status (FMs with AD vs. FMs without AD), the number, crude proportions (%), and age-adjusted proportions (ratios per 1000 FMs) of FMs for each variable of interest. Among the 1,036,069 FMs available to the TRACTOR project, 5067 individuals had AD. The median follow-up was 1563 (1058–1826) days, with a total of 3,135,093 person-years, and 1.62 [1.57–1.66] AD cases per 1000 person-years. For females, there were 846,523 person-years, with 3.61 [3.48–3.74] cases per 1000 person-years, while for males, there were 2,288,570 person-years, with 0.88 [0.84–0.92] cases per 1000 person-years. When considering only FMs who were 60 years or older (SA2), there were 18.25 [17.70–18.80] AD cases per 1000 person-years, 25.52 [24.56–26.48] female AD cases per 1000 person-years, and 11.98 [11.37; 12.59] male AD cases per 1000 person-years. Among FMs who were 60 years or older, there were also 19.41 [18.83–19.99] ADRD cases per 1000 person-years, 26.93 [25.92–27.93] female ADRD cases per 1000 person-years, and 12.81 [12.16; 13.46] male ADRD cases per 1000 person-years. Regarding the 5067 AD cases (main analysis), 1960 FMs (39%) had solely an AD insurance declaration (LTI) while 10% were identified solely based on drug prescriptions, and 51% were both identified with an AD insurance declaration and drug prescription. Table S3 provides the number of identified cases for each sensitivity analysis. There were between 2609 identified cases for SA7 and 5430 for SA3, respectively.

**Table 2:** Baseline characteristics of the study population, TRACTOR project, France, 2002–2016

Characteristics	FMs without AD (n = 1,031,002) No. (%) [age-adjusted ratio per 1000 FMs]	FMs with AD (n = 5067) No. (%) [age-adjusted ratio per 1000 FMs]
*Sex*		
Female	317,334 (31) [40]	3058 (60) [50]
Male	713,668 (69) [90]	2009 (40) [80]
Age (years), mean (SD)	46.5 (14.1)	68.9 (8.9)
*Family status*		
Single	391,732 (38) [55]	1823 (36) [47]
As a couple	639,270 (62) [74]	3244 (64) [83]
*First year of the farm’s establishment*		
before 1985	105,316 (10) [18]	771 (15) [23]
1985–1994	437,238 (42) [63]	3173 (63) [79]
1995–2004	254,401 (25) [32]	965 (19) [34]
after 2004	238,884 (23) [30]	166 (3.3) [12]
*Median yearly farm surface (hectares)*		
Farm surface = 0 hectares	109,895 (10) [14]	78 (1.5) [14]
0 < farm surface < 5 hectares	226,739 (22) [29]	2784 (55) [30]
5 ≤ farm surface < 25 hectares	268,566 (26) [34]	1194 (24) [31]
25 ≤ farm surface < 50 hectares	197,291 (19) [25]	541 (11) [33]
Farm surface ≥ 50 hectares	254,254 (25) [32]	529 (10) [36]
*Number of main activities performed between 2002 and 2016*		
Performed only one main activity	931,754 (90) [117]	4560 (90) [121]
Performed two main activities	93,414 (9.1) [12]	484 (9.6) [11]
Performed 3 or more main activities	5834 (0.6) [0.7]	23 (0.5) [0.3]
*Farm location (geographical area)*		
Auvergne-Rhône-Alpes	111,860 (11) [14]	580 (11) [14]
Bourgogne-Franche-Comté	62,955 (6.1) [7.9]	270 (5.3) [9.1]
Bretagne	77,048 (7.5) [9.7]	192 (3.8) [13]
Centre – Val de Loire	47,449 (4.6) [6.0]	204 (4.0) [5.3]
Corse	5055 (0.5) [0.6]	4 (0.1) [0.4]
Grand Est	79,236 (7.7) [9.9]	263 (5.2) [14]
Hauts-de-France	45,910 (4.5) [5.8]	194 (3.8) [5.0]
Île-de-France	12,991 (1.3) [1.6]	49 (1.0) [2.0]
Normandie	76,360 (7.4) [9.6]	574 (11) [11]
Nouvelle-Aquitaine	170,893 (17) [21]	931 (18) [23]
Occitanie	157,291 (15) [20]	1016 (20) [30]
Provence-Alpes-Côte d’Azur	105,268 (10) [13]	550 (11) [18]
Pays de la Loire	78,686 (7.6) [9.9]	240 (4.7) [8.3]
*Number of farms*		
1 farm	1,005,473 (98) [126]	5003 (99) [127]
> 1 farm	25,259 (2.4) [3.2]	64 (1.2) [3.9]
*Partner work status*		
Do not perform task to help farm manager	906,818 (88) [114]	4796 (95) [111]
Perform task to help farm manager	124,184 (12) [16]	271 (5.0) [25]
*Number of associates*		
No associate	772,871 (75) [98]	4619 (91) [101]
At least one associate	258,131 (25) [31]	448 (9.0) [33]
*Median yearly insurance premiums (euros)*		
insurance premiums = 0 euro	39,353 (3.8) [4.9]	130 (2.6) [6.3]
0 < insurance premiums < 1500 euros	270,655 (26) [34]	3012 (59) [27]
1500 ≤ insurance premiums < 5000 euros	216,384 (21) [27]	888 (18) [38]
5000 ≤ insurance premiums < 10,000 euros	245,052 (24) [31]	534 (11) [41]
Insurance premiums ≥ 10,000 euros	303,732 (29) [38]	604 (12) [44]
*Secondary activity*		
No secondary activity	655,837 (64) [82]	4670 (92) [113]
At least one secondary activity	375,165 (36) [47]	397 (8.0) [19]
*Unemployment status*		
Never unemployed	1,030,966 (99) [129]	5067 (100) [130]
Had been unemployed at least once	621 (0.1) [0.4]	0 (0) [0]
*Retirement status*		
FMs who did not retire between 2002 and 2016	761,604 (73) [95]	1464 (28) [75]
FMs who retired between 2002 and 2016	274,465 (27) [46]	3603 (71) [87]
*Number of pre-existing medical comorbidities*		
0 comorbidity	678,725 (66) [84]	2400 (47) [9.2]
1 comorbidity	171,137 (17) [26]	1439 (28) [30]
> 1 comorbidity	181,140 (18) [19]	1228 (24) [92]

AD, Alzheimer’s disease; FM, farm manager; No, number; SD, arithmetic standard deviation

The proportion of females was higher among FMs with AD than without (60 vs. 31%). There were 274,465 (26.5%) FMs who retired between 2002 and 2016, with a higher proportion among FMs with AD than without (71.1 vs. 26.3%). Overall, FMs with AD were older than FMs without AD (mean age of 69 years old versus 47 years old), established their farm in earlier time periods (average year of 1989 vs. 1995), had smaller farm surfaces (median of 4.4 hectares vs.17.3), a higher number of comorbidities, paid lower insurance premiums (median of 940 euros per year vs. 5257), worked more often in South of France, and less of them had secondary activity (8 vs. 36%) and associates (5 vs. 12%).

AD affected more females (60%) than males (40%). Most FMs with AD had a F00 ICD-10 code (51%), followed by a F03 code (29%), and a G30 code (4%) (Table S4). Some FMs with an AD drug (n = 71, 1.4%) had both a LTI for AD and PD. Most FMs with AD were prescribed memantine (24%), followed by rivastigmine (18%), donepezil (13%), and galantamine (5%) (Table S4). No FM was prescribed with N06DA52 or N06DA53. FMs with AD were prescribed most of the time with only one type of drug (Table S4). Most AD cases were found in crop farming (56%), followed by viticulture (14%), dairy farming (11%), cow farming (9%), and unspecified and mixed farming (7%) (Table 3 and Table S5). No FM whose first exposure year occurred during the follow-up period (2012–2016) was diagnosed with AD.

**Table 3:** Risks of Alzheimer’s disease by agricultural activity, TRACTOR project, France, 2002–2016

Agricultural activity	Sex	Study population No. (%)	AD^*^ No. (%)	Unexposed AD No.	HR (95% CI)^†^	HR (95% CI)^‡^
Truck farming, floriculture/flower-growing	Both sexes	41,525 (4.0)	149 (2.9)	4918	0.93 (0.78–1.09)	1.02 (0.87–1.19)
	Female	12,672 (4.0)	92 (3.0)	2966	1.10 (0.89–1.35)	1.07 (0.68–1.68)
	Male	28,853 (4.0)	57 (2.8)	1952	**0.75 (0.57–0.98)**	0.90 (0.70–1.16)
Fruit arboriculture	Both sexes	24,086 (2.3)	137 (2.7)	4930	**1.36 (1.15–1.62)**	**1.24 (1.05–1.47)**
	Female	7649 (2.4)	77 (2.5)	2981	**1.28 (1.02–1.61)**	**3.45 (1.27–9.38)**
	Male	16,437 (2.3)	60 (3.0)	1949	**1.37 (1.06–1.78)**	**1.31 (1.01–1.69)**
Garden center/tree nursery	Both sexes	5111 (0.5)	12 (0.2)	5055	0.63 (0.36–1.11)	0.69 (0.39–1.21)
	Female	1358 (0.4)	6 (0.2)	3052	Not calculated	Not calculated
	Male	3753 (0.5)	6 (0.3)	2003	Not calculated	Not calculated
Crop farming (e.g. wheat, corn, and industrial grower)	Both sexes	305,838 (30)	2837 (56)	2230	**3.72 (3.47–3.98)**	**2.60 (2.46–2.75)**
	Female	102,240 (32)	1789 (59)	1269	**4.15 (3.80–4.54)**	**3.38 (2.27–5.04)**
	Male	203,598 (28)	1048 (52)	961	**2.85 (2.55–3.19)**	**2.47 (2.26–2.70)**
Viticulture	Both sexes	118,577 (11)	723 (14)	4344	**1.29 (1.18–1.42)**	**1.12 (1.03–1.21)**
	Female	41,970 (13)	447 (15)	2611	**1.29 (1.15–1.46)**	**2.41 (1.32–4.39)**
	Male	76,607 (11)	276 (14)	1733	**1.73 (1.49–2.02)**	**1.24 (1.09–1.40)**
Unspecified specialized farming (e.g. herbs, mushrooms)	Both sexes	6168 (0.6)	11 (0.2)	5056	0.73 (0.40–1.32)	0.56 (0.31–1.00)
	Female	2233 (0.7)	6 (0.2)	3052	Not calculated	Not calculated
	Male	3935 (0.6)	5 (0.3)	2004	Not calculated	Not calculated
Dairy farming	Both sexes	158,706 (15)	534 (11)	4533	**0.67 (0.61–0.73)**	**0.82 (0.75–0.90)**
	Female	48,823 (15)	306 (10)	2752	**0.65 (0.57–0.73)**	1.38 (0.92–2.08)
	Male	109,883 (15)	228 (11)	1781	0.89 (0.77–1.03)	0.87 (0.76–1.00)
Cow farming	Both sexes	110,214 (11)	459 (9.1)	4608	**0.84 (0.76–0.93)**	**0.76 (0.69–0.84)**
	Female	32,699 (10)	281 (9.2)	2777	0.89 (0.78–1.01)	0.71 (0.41–1.23)
	Male	77,515 (11)	178 (8.9)	1831	**0.68 (0.58–0.80)**	**0.78 (0.67–0.90)**
Both/mixed dairy and cow farming	Both sexes	30,729 (3.0)	79 (1.6)	4988	**0.46 (0.37–0.57)**	**0.53 (0.42–0.66)**
	Female	8004 (2.5)	44 (1.4)	3014	**0.48 (0.36–0.65)**	1.19 (0.53–2.68)
	Male	22,725 (3.2)	35 (1.7)	1974	**0.57 (0.40–0.79)**	**0.52 (0.37–0.73)**
Ovine and caprine farming	Both sexes	47,086 (4.5)	104 (2.1)	4963	**0.50 (0.41–0.61)**	**0.53 (0.44–0.63)**
	Female	16,808 (5.3)	58 (1.9)	3000	**0.54 (0.42–0.71)**	**0.38 (0.16–0.92)**
	Male	30,278 (4.2)	46 (2.3)	1963	**0.65 (0.48–0.87)**	**0.62 (0.46–0.82)**
Pig farming	Both sexes	13,389 (1.3)	13 (0.3)	5054	**0.30 (0.18–0.52)**	**0.27 (0.16–0.45)**
	Female	3830 (1.2)	5 (0.2)	3053	Not calculated	Not calculated
	Male	9559 (1.3)	8 (0.4)	2001	Not calculated	Not calculated
Stud farming	Both sexes	15,641 (1.5)	39 (0.8)	5028	1.25 (0.91–1.71)	**0.68 (0.49–0.93)**
	Female	6831 (2.1)	25 (0.8)	3033	1.30 (0.87–1.93)	0.82 (0.30–2.23)
	Male	8810 (1.2)	14 (0.7)	1995	1.02 (0.60–1.73)	0.59 (0.35–1.00)
Training, dressage, riding clubs	Both sexes	13,273 (1.3)	11 (0.2)	5056	0.68 (0.38–1.23)	**0.49 (0.27–0.88)**
	Female	6049 (1.9)	3 (0.1)	3055	Not calculated	Not calculated
	Male	7224 (1.0)	8 (0.4)	2001	Not calculated	Not calculated
Unspecified large animal farming (e.g. ostrich, llama)	Both sexes	2663 (0.3)	3 (0.1)	5064	Not calculated	Not calculated
	Female	1280 (0.4)	3 (0.1)	3055	Not calculated	Not calculated
	Male	1383 (0.2)	0	2009	Not calculated	Not calculated
Poultry and rabbit farming	Both sexes	24,576 (2.4)	24 (0.5)	5043	**0.29 (0.20–0.44)**	**0.28 (0.19–0.41)**
	Female	9671 (3.0)	13 (0.4)	3045	**0.25 (0.14–0.43)**	1.58 (0.51–4.94)
	Male	14,905 (2.1)	11 (0.6)	1998	**0.36 (0.20–0.66)**	**0.37 (0.20–0.66)**
Unspecified small animal farming (e.g. frogs, snails, bees)	Both sexes	18,058 (1.7)	15 (0.3)	5052	0.66 (0.40–1.10)	**0.35 (0.21–0.58)**
	Female	7698 (2.4)	7 (0.2)	3051	Not calculated	Not calculated
	Male	10,360 (1.5)	8 (0.4)	2001	Not calculated	Not calculated
Unspecified and mixed farming (e.g. polyculture, mixed farming, diversified farming)	Both sexes	120,746 (12)	370 (7.3)	4697	**0.70 (0.63–0.78)**	**0.63 (0.57–0.70)**
	Female	36,955 (12)	208 (6.8)	2850	**0.62 (0.54–0.72)**	0.94 (0.50–1.74)
	Male	83,791 (12)	162 (8.1)	1847	**0.78 (0.66–0.94)**	**0.71 (0.61–0.82)**
Shellfish farming (e.g. oyster farming, scallop aquaculture)	Both sexes	3350 (0.3)	5 (0.1)	5062	Not calculated	Not calculated
	Female	666 (0.2)	1 (0.03)	3057	Not calculated	Not calculated
	Male	2684 (0.4)	4 (0.2)	2005	Not calculated	Not calculated
Salt works/salt evaporation pond	Both sexes	873 (0.1)	2 (0.04)	5065	Not calculated	Not calculated
	Female	200 (0.1)	2 (0.1)	3056	Not calculated	Not calculated
	Male	673 (0.1)	0	2009	Not calculated	Not calculated
Sylviculture/forestry (e.g. thinning, pruning)	Both sexes	1986 (0.2)	10 (0.2)	5057	1.19 (0.64–2.21)	1.52 (0.84–2.75)
	Female	339 (0.1)	7 (0.2)	3051	Not calculated	Not calculated
	Male	1647 (0.2)	3 (0.2)	2006	Not calculated	Not calculated
Wood production (e.g. lopping)	Both sexes	10,470 (1.0)	7 (0.1)	5060	Not calculated	Not calculated
	Female	283 (0.1)	1 (0.03)	3057	Not calculated	Not calculated
	Male	10,187 (1.4)	6 (0.3)	2003	Not calculated	Not calculated
Stationary sawmill (e.g. edging, trimming, decking, debarking)	Both sexes	735 (0.1)	3 (0.1)	5064	Not calculated	Not calculated
	Female	48 (0.02)	0	3058	Not calculated	Not calculated
	Male	687 (0.1)	3 (0.2)	2006	Not calculated	Not calculated
Agricultural work companies (e.g. pesticide applications, harvest reaping)	Both sexes	14,282 (1.4)	17 (0.3)	5050	0.67 (0.41–1.08)	**0.60 (0.37–0.97)**
	Female	1715 (0.5)	6 (0.2)	3052	Not calculated	Not calculated
	Male	12,567 (1.8)	11 (0.6)	1998	**0.40 (0.22–0.72)**	**0.49 (0.27–0.89)**
Gardening, landscaping and reforestation companies	Both sexes	44,948 (4.3)	30 (0.6)	5037	0.96 (0.65–1.42)	0.78 (0.55–1.11)
	Female	2369 (0.7)	4 (0.1)	3054	Not calculated	Not calculated
	Male	42,579 (6.0)	26 (1.3)	1983	0.88 (0.57–1.37)	**0.63 (0.43–0.92)**
Rural craftsperson (e.g. mason, mechanics)	Both sexes	7038 (0.7)	3 (0.1)	5064	Not calculated	Not calculated
	Female	256 (0.1)	1 (0.03)	3057	Not calculated	Not calculated
	Male	6782 (1.0)	2 (0.1)	2007	Not calculated	Not calculated
Company representative/authorized representative	Both sexes	1846 (0.2)	0	5067	Not calculated	Not calculated
	Female	1437 (0.5)	0	3058	Not calculated	Not calculated
	Male	409 (0.1)	0	2009	Not calculated	Not calculated

Bold values refer to hazard ratios that do not include unity (one) in their confidence intervals

AD, Alzheimer’s disease; HR, hazard ratio; m, number of exposed AD cases

*The percentages in brackets refer to the ratio of exposed AD cases in the study population, and the total number of AD cases in the overall population

^†^Main analysis: adjusted for sex (for “both sexes” only), age, the first year of the farm’s establishment, farm surface, earnings, number of associates, unemployment status, total number of farms, family status, partner work status, farm location, number of comorbidities, and having a secondary activity

^‡^Sensitivity analysis 1: adjusted for sex (for “both sexes” only), age, and the first year of the farm’s establishment for all models

### AD risk associated with agricultural activities

**Fig. 1:**
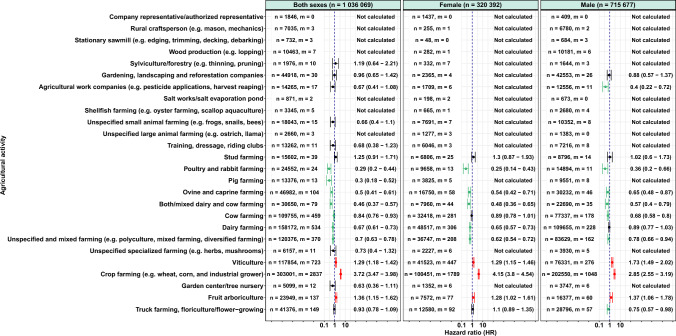
Agricultural activities and risks of AD, TRACTOR project, France, 2002–2016. Multivariable Cox regression models for Alzheimer’s disease according to each agricultural activity (y-axis) are displayed when the number of exposed cases was sufficient (m ≥ 10). The hazard ratio is represented by a point (x-axis) while error bars represent the 95% confidence interval. The red error bars refer to a higher risk of Alzheimer’s disease, while the green error bars represent a lower risk of Alzheimer’s disease. The black error bars indicate situations where there is no difference in risk of Alzheimer’s disease among the farm managers performing the considered activity compared to the population of farm managers not performing the considered activity. n, number of exposed farm managers; m, number of exposed AD cases; HR, hazard ratios (95% confidence interval); adjusted for sex (for “both sexes” only), age, first year of the farm’s establishment, farm surface, insurance premiums, number of associates, unemployment status, total number of farms, family status, partner work status, farm location, number of comorbidities, and having a secondary activity

Associations varied by sex as well as types of crops and animal farming (Fig. 1 and Table 3). Crop farming was associated with the highest risks of AD (HR = 3.72 [3.47–3.98]), which were higher for females (HR = 4.15 [3.80–4.54]) than for males (HR = 2.85 [2.55–3.19]). Viticulture (HR = 1.29 [1.18–1.42]) and fruit arboriculture (HR = 1.36 [1.15–1.62]) were also associated with higher risks of AD, with higher risks for males than females.

**Fig. 2:**
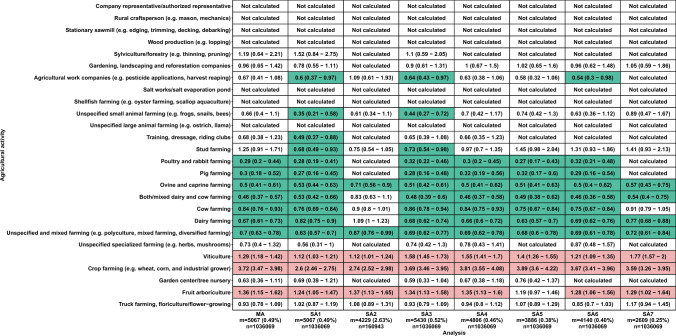
Agricultural activities and risks of AD, TRACTOR project, France, 2002–2016. Comparison of the main analysis with the sensitivity analyses. Multivariable Cox regression models for Alzheimer’s disease according to different analyses (x-axis) for which each agricultural activity (y-axis) are displayed when the number of exposed cases was sufficient (m ≥ 10). The hazard ratio is provided with a 95% confidence interval for each analysis. The red cells refer to a higher risk of Alzheimer’s disease, while the green cells represent a lower risk of Alzheimer’s disease. The white cells indicate situations where there is no difference in risk of Alzheimer’s disease among the farm managers performing the considered activity compared to the population of farm managers not performing the considered activity. m, total number of cases; MA, main analysis; n, total number of FMs; SA, sensitivity analysis; MA, adjusted for sex (for “both sexes” only), age, first year of the farm’s establishment, farm surface, earnings, number of associates, unemployment status, total number of farms, family status, partner work status, farm location, number of comorbidities, and having a secondary activity; SA1, adjusted for sex (for “both sexes” only), age, and the first year of the farm’s establishment for all models. SA2: included only FMs who were 60 years and older, and adjusted for the same variable as the main analysis; SA3, included FMs diagnosed with AD and other related dementias, and adjusted for the same variable as the main analysis; SA4, adjusted for the same variable as the main analysis, but FMs who were prescribed with an AD drug that also had a LTI declaration for PD were not considered as AD cases; SA5, adjusted for the same variable as the main analysis, but FMs who were prescribed with an AD drug that also had a LTI declaration for PD or a LTI for unspecified dementia were not considered as AD cases; SA6, adjusted for the same variable as the main analysis, but the AD case identification was restricted to LTI declarations; SA7, adjusted for the same variable as the main analysis, but the AD case identification was restricted to drug prescriptions

By contrast, analyses showed several lower risks of AD, in particular for several animal farming types. Poultry and rabbit farming (HR = 0.29 [0.20–0.44]), ovine and caprine farming (HR = 0.50 [0.41–0.61]), unspecified and mixed farming (HR = 0.70 [0.63–0.78]), mixed dairy and cow farming (HR = 0.46 [0.37–0.57]), dairy farming (HR = 0.67 [0.61–0.73]), and pig farming (HR = 0.30 [0.18–0.52]) were associated with lower risks of AD, for both females and males, with risks always lower for females than for males. Cow farming was associated with lower risks of AD, but with a higher risk in females (HR = 0.89 [0.78–1.01]) than in males (HR = 0.68 [0.58–0.80]). Agricultural work companies, and truck farming, floriculture/flower-growing were associated with lower risks of AD, but only for males (HR = 0.40 [0.22–0.72] and HR = 0.75 [0.57–0.98], respectively). All sensitivity analyses yielded similar results to the main analysis (Figs. 2, S1, S2, and Table S5). However, there were fewer activities with lower risks for SA3 (analysis restricted to FMs aged 60 years or more), but the number of FMs studied for this sensitivity analysis was far smaller than for other analyses (160,943 vs. 1,036,069) (Table S3).

## Discussion

In this study, higher and lower risks of AD were observed in relation to several agricultural activities performed by French FMs. Associations varied with kinds of crop and animal farming, suggesting that part of the risk could be potentially attributable to occupational agricultural activities. In addition, for a given agricultural activity, associations could vary by sex, suggesting that differences in occupational exposure and tasks, sometimes sex-specific, could contribute to the differences in risks between females and males.

### Characteristics of FMs with AD

FMs with AD were older than FMs without AD, which is not surprising because AD affects individuals late in life, usually starting after 60 years old [7]. AD affected more females (60%) than males (40%), which is in accordance with observations from the general population, where 66% of AD patients are females [33]. Compared to FMs without AD, AD FMs had considerably smaller farm surfaces and earnings. One possible explanation could be that AD FMs were much older than FMs without AD, implying that they may be less active and less productive, in particular because AD is characterized by a progressive decline in thinking leading to work disabilities [5, 10]. This hypothesis would imply that AD FMs handed on a part of their farm because of AD progression, which is something that we cannot assess or know with the data at our disposal.

FMs who were 60 years or older had a higher incidence of AD than individuals from the French general population (18.25 vs. 11.0 cases per 1000 person-years) [47]. The incidence of ADRD in FMs who were 60 years or older (19.41 cases per 1000 person-years) was slightly higher than those from the French general population (18.2 cases per 1000 person-years), and in high-income countries for persons over 60 years (18.39 cases per 1000 person-years) [47].

### Possible risk factors

AD is a complex and multifactorial disorder involving many risk factors. Several possible risk factors have been identified, such as aging, smoking, low education and/or low occupational complexity, obesity, diabetes, midlife hypertension, physical inactivity, infections, and environmental pollutants such as occupational exposure to pesticides [1, 7, 8, 11, 24, 48]. Regarding pesticide exposure (a possible risk factor), results are inconsistent. Several studies found no evidence or weak evidence of association between pesticide exposure and the risk of AD and other dementia-related outcomes (e.g. cognition, brain health) [9, 23, 25, 26, 29, 49, 50], with only a few that reported higher risks of AD [9, 38, 40, 51]. Several reviews and meta-analyses support an association between exposure to pesticides and cognitive dysfunction, dementia, and AD, with the strongest evidence for occupational exposure [23, 25, 26, 29]. A recent review suggested “rather strong” evidence [25], while two meta-analyses reported positive associations, with one based on four studies (weighted RR = 1.50 [0.98–2.29]) [26], and the other one based on seven studies (weighted RR = 1.34 [1.08–1.67]) [23]. In addition, a French collective expert review concluded there was a moderate link between exposure to pesticides and the risk of AD [34]. The European Environment Agency mentions suspected evidence [52], but the UK Parliamentary Office of Science and Technology did not suggest this association [53].

### Risks associated with agricultural activities

Some FMs might be exposed to one or several of the aforementioned possible risk factors, in particular social isolation, low educational status, exposure to excessive noise, as well as exposure to pesticides, solvents, and some inorganic elements belonging to metal and metalloid families [2, 6, 7, 25, 34, 35]. Contrary to PD, data from the literature is scarce and lacking regarding the risk of AD for farmers [37–40]. To the best of our knowledge, we found no studies reporting either a lower or higher risk of AD related to specific agricultural activities.

Our analyses showed higher risks of AD for crop farming, viticulture, and fruit arboriculture, with higher risks for males than females, with the exception of crop farming, suggesting potential sex-specific tasks and exposure, as already pointed out in the literature [54]. Besides sex-specific tasks, other possible factors could potentially explain the observed sex differences regarding the risk of AD. Some studies suggest that males are more susceptible to OP pesticides [55], that they use more pesticides than females, but that fewer of them apply protective measures or behaviors when using pesticides [56]. In addition, females have a higher prevalence of depression than males [57–59], which is associated with an increase in the risk of developing dementia [35].

In our study population, AD FMs performed more often activities where pesticides are known to be used than FMs without AD (81 vs. 62%) [60, 61]. In 2006, crop farming was the activity for which the pesticide used in France was the highest (67% of all pesticides used), followed by viticulture (15%), and fruit arboriculture (5.2%) [60]. Some studies have shown that pesticide levels are higher in the farming population than in other occupations or in the general population, with, for instance, higher urinary and blood concentrations of chlorpyrifos’s metabolite, glyphosate, or pyrethroid [62–66].

For crop farming, viticulture, and fruit arboriculture, all FMs are exposed to pesticides to fight against pests and insects, and prevent plant diseases, in particular neurotoxic insecticides such as OPs or pyrethroids [67–69]. The occupational exposure to pesticides could play a role in the positive associations observed in this study, as suggested by the growing literature. A longitudinal population-based study in Canada reported an increased risk of AD for occupational exposure to fumigants and defoliants (adjusted RR = 4.35 [1.05–18]) [40]. Another Canadian study found a higher risk of AD for occupational exposure to unspecified pesticides (OR = 2.17 [1.18–3.99]) [9]. A community-based study conducted in the US, including individuals aged ≥ 65 years, reported higher risks of AD among pesticide-exposed individuals (HR = 1.42 [1.06–1.91]), for OP (HR = 1.53 [1.05–2.23]), and for organochlorine (HR = 1.49 [0.99–2.24]) exposures [38]. A large prospective cohort study (PAQUID) conducted in France, including 1507 elderly participants aged ≥ 65 years, reported higher risks of AD for occupationally exposed males (RR = 2.39 [1.02–5.63] vs. RR = 0.89 [0.49–1.62] for females), using information from a job exposure matrix. However, this study found no increased risks for the main job in agriculture (RR = 1.32 [0.43–4.10] for males vs. RR = 0.85 [0.40–1.86] for females) [37]. FMs can also be exposed to inorganic elements that might play a role in neurodegeneration and potentially explain some of our findings [13–18, 20, 24–26, 29]. Several inorganic compounds are or have been used by farmers. Many fertilizers used in agriculture contain known or suspected neurotoxic heavy metals, in particular cadmium, lead, zinc, and copper [17, 70, 71]. Several fungicides contain neurotoxic metals, such as manganese and zinc, as well as mercury, that have been used in the past [29, 71]. For instance, dithiocarbarmates are a class of fungicides that have been widely used to control fungal pathogens in a broad range of crops since the 40s [30]. Several of these fungicides contain metals, in particular maneb that contains manganese, ziram and zineb that contain zinc and were used in fruit arboriculture and crop farming, or mancozeb that contains both manganese and zinc [30, 71]. Copper, which is a suspected neurotoxic compound [15, 16, 20, 29], is largely used in agriculture as a fertilizer and as a fungicide (25% CuSO_4_ in the Bordeaux mixture), in particular in vineyards and in organic farming [49, 72]. Organic mercury compounds, such as methylmercury, were extensively used as fungicides in the past for the prevention of seed-borne diseases in grains and cereals [30]. Manganese, known as a neurotoxic compound, is also commonly used in fertilizers and is present in some carbamate fungicides such as maneb or mancozeb that are used in crop farming and fruit arboriculture [14–16, 20, 24, 29, 73]. Finally, arsenic is a metalloid that was historically involved in agriculture (e.g. viticulture, crop farming) as an insecticide and then only as a fungicide until its complete withdrawal [13–16, 18, 19, 25, 29]. Pesticides naturally derived from arsenic, such as rotenone and pyrethrum, were also used, in particular in crop farming, viticulture, and fruit arboriculture [14]. Depending on the activity and farming approach (conventional or organic), the pesticides used can differ [74–76]. However, in our study, no specific pesticide class can be pinpointed due to the lack of available information on the use of specific pesticides for the studied activities. Because the type of farming approach used (conventional or organic) was not known, it was also not possible to study the impact of the farming approach on AD risk. Even though the amount and frequency of pesticides used are the highest for crop farming, viticulture, and fruit arboriculture, pesticides are also used in other agricultural activities such as truck farming, agricultural work companies, gardening, landscaping and reforestation companies, and animal husbandry, in particular for sheep dip [9, 37]. For these activities, the use of pesticides, both in terms of amount and frequency, is smaller than for crop farming, viticulture, and fruit arboriculture. In addition, the nature of the pesticides used (not the same pests and diseases) and the mode of application differ between activities, with a higher probability of exposure for crop farming, viticulture, and fruit arboriculture compared to other activities [9, 63, 77, 78]. Regarding sheep dip, no association between dementia and low chronic pesticide exposure in sheep farmers has been suggested so far [9, 79]. To the best of our knowledge, no previous studies have investigated the risk of AD or other related dementias in animal farming. Our analyses showed lower risks of AD for all animal-farming types, with the exception of stud farming and unspecified animal farming. Risks were always lower for females than for males, with the exception of cow farming, suggesting potential sex-specific tasks. There are several differences in the nature of tasks performed (Table S1) between animal farming activities and agricultural activities that were found to have a higher risk of AD. These differences could play a role and potentially explain some of the risk differences observed. Contrary to viticulture, crop farming, and fruit arboriculture, the pesticide exposure levels for FMs involved in animal husbandry are low or very low. Some studies have shown that farmers involved in animal farming are associated with more intense physical activity levels than crop farmers or activities involving a more frequent use of machinery [80–83]. Given that physical activity is a possible protective factor for AD [1–3, 5, 7, 12, 84], it could potentially play a role in the differences we observed. The same hypothesis could also applied to other activities such as gardening, landscaping and reforestation companies for which the physical activity levels can be high (e.g. chainsaw and skidder operations, climbing on trees). In addition, for crop farming, viticulture, and fruit arboriculture, farmers can frequently be exposed to excessive noise from grain dryers, tractors, combines, and other powered equipment [35]. Farmers are also reluctant to wear hearing protection devices [35]. Excessive noise can sometimes lead to hearing loss, which is a possible risk factor for AD. Studies have shown that agricultural workers are more likely to experience noise-induced hearing loss than workers in other occupational settings [35]. Several studies have also reported that human-animal interactions have many beneficial psychosocial and psychophysiological effects on people with and without medical health conditions, in particular on pet owners [85, 86]. It has also been shown that systolic blood pressure and plasma triglyceride levels are lower when compared with non-owners of animals [85].

### Strengths and limitations

The most important strengths of our study are the large number of exposed cases, the completeness of the available data, and the detail about agricultural activities. To lessen the possibility of chance findings, we conducted an analysis only when the number of exposed cases was ≥ 10. In addition, results from the sensitivity analysis adjusting only on sex, age, and the first year of the farm’s establishment were similar to the findings from the analysis that adjusted on more variables. False associations resulting from multiple comparisons might be an issue, but approaches used to limit false positive findings (type I errors) are too conservative, increase the risk of false negative findings (type II errors), and are not relevant in the framework of a large cohort study with data on multiple illnesses [87]. The Benjamini–Hochberg approach was used to account for multiple testing. However, it is not straightforward to choose the appropriate familywise test, the most relevant type I error threshold, and multiple comparison adjustments could hamper the discovery of an effect worthy of further study, which is why we also provided unadjusted *p*-values [88].

One of the main limitations of this work is that the data available from the TRACTOR project does not allow us to include FM who retired before 2002. However, all FMs who worked at least one year between 2002 and 2016, and who retired during that time period were included. Retirement was not a cause of loss of follow-up (right censor), so there should not be a competing risk associated with retirement. The retirement status (active or retired) was not added to the model since this variable was highly correlated with age. Due to the left-censored nature of the data, for FMs who retired in the first years of the study period (2002–2016), only one (if they retired in 2003) to a couple of years of exposure was taken into account, which could bias risk estimation. It is, however, impossible to know the magnitude of this bias.

Although AD is the most common form of dementia, its diagnosis remains complex and can be confused with other types of dementia, especially during the early stages of the disease [89, 90]. In addition, determining the exact type of dementia remains difficult, as symptoms and brain damage associated with different forms of dementia overlap. Formal AD diagnosis is now strengthened by the use of biomarkers (analysis of amyloid and tau protein in the cerebrospinal fluid and/or amyloid positron emission tomography), even if it can only be confirmed by postmortem brain autopsy [91, 92]; none of which were available for the study. In addition, detection of AD is limited by the difficulties of recognition by the family or GP, the complexity of diagnosis, and the presence of comorbidities that alter a proper diagnosis. Several studies suggest that the number of AD patients is underestimated, with about 1 in 2 to 3 cases being undiagnosed [40, 93, 94]. In our study, AD cases were identified either using ICD-10 codes assigned to each FM that benefits from health care expenditure coverage for AD as part of the LTI insurance declaration scheme or with ATC codes corresponding to the prescription of drugs used in AD. According to Gallini et al. [95], no consensus exists about the best algorithm to identify AD using administrative health databases. Using both ICD-10 and ATC codes allowed us to better identify AD cases, even though it is not ideal, both in terms of sensitivity and specificity. Regarding ICD-10 codes, LTI declaration is not automatic because the GP may consider the request unnecessary, in particular since there is a lack of effective treatments [96]. Regarding ATC codes, drugs used in AD are mostly specific [5, 48, 51], with the exception of rivastigmine, which is extended to other indications beyond AD, including PD. To address potential misidentification, several more restricted sensitivity analyses were conducted. These sensitivity analyses yielded similar results to the main analysis, which indicates that misidentification should be limited. However, for all of the aforementioned reasons, we cannot exclude possible cases of misclassification and underestimation. The identified AD cases within this insurance health database reflect the AD cases supported by the French healthcare system but not the entire French farming population suffering from AD. Therefore, both ICD-10 and ATC codes are not comparable to the real AD incidence and could misestimate risk estimation. It is, however, difficult to assess the direction of errors or biases.

Any FM who did not experience the event of interest (AD diagnosis) during the follow-up period was treated as censored (right censoring), which is a limitation. Indeed, given the age distribution of the study participants, death could represent a competing risk (i.e. mortality precluding AD onset). Because the vital status was not available in MSA data, we cannot exclude the presence of the competing risk of death. Not accounting for this competing risk could lead to an overestimation of the cumulative incidence of the event of interest [97]. To address this bias, a perspective from this work would be to link MSA data with the French national registry of specific causes of mortality (CépiDC) to identify FMs who died during the follow-up period and obtain their cause of death [98]. With the vital status known, the competing risk of death could then be considered by using a multi-state model [99].

Confounding factors not available to TRACTOR could represent a bias, but their potential impact on the results is hard to evaluate because these variables were not available [100]. It is possible that their absence could bias the estimated effects and confound or mask the genuine relationship between agricultural activities and AD. In addition, regarding available confounders, their accuracy was sometimes limited, so the possibility of residual confounding cannot be excluded. Although information on chemical agents used by FMs and several potential confounders (e.g. smoking, education level) were not available due to the inherent nature of the available data (insurance health database), risks were adjusted for important confounders (sex, age, geographical area, medical comorbidities) and on several covariates after a conservative selection. Only an indirect exposure estimation was possible using activities from administrative databases, with no information on chemical, physical, or biological agents that could be encountered/used by FMs. Some activities were not descriptive enough to provide the best risk estimation possible, in particular for activities that are highly heterogeneous in nature, such as crop farming or agricultural work companies, for which it is difficult to speculate the exposures of interest. Hence, there could be differences in exposures within activities. In addition, the reference group included FMs who did not carry out the activity of interest. This could have the benefit of comparing a group more similar to the exposed than the general population. However, job title could be a proxy for exposures associated with AD that occur in those who engage in the activity and those who do not (for example, pesticide exposure could occur in several of the activities studied). Therefore, the association of the activity may not reflect the association of the underlying exposures for which it is a proxy. In addition, only information about the main yearly activity in terms of effective working time was known. Thus, some FMs in the reference group may have carried out the activity studied but not as their main activity, which is a limitation. To address the bias introduced by co-exposures, analyses were adjusted on secondary activities but not on past main activities because most FMs (90%) never changed their main activity between 2002 and 2016. No specific pesticide class or chemical can be pinpointed due to the lack of available information on the use of these substances. To refine the analysis and address the aforementioned issues, external sources (e.g. cohort studies and exposure matrices) could be linked to TRACTOR. In addition, further studies (e.g. qualitative, quantitative, or mixed-methods studies) regarding crop farming, viticulture, and fruit arboriculture are needed to understand and identify which factors contribute to the observed risks. Because agricultural practices and risk factors can differ between countries, the generalizability of our findings could be limited.

## Conclusions

Results from this work bring new insights and shed some light on the association between a wide range of agricultural activities and the risk of AD in FMs, overall and for both sexes. The findings suggest that the highest risks of AD were found in agricultural activities where the use of pesticides is the highest [60, 61], namely crop farming, viticulture, and fruit arboriculture, while the lowest risks were found among breeders. These findings highlight the necessity of expanding the research on identifying the determinant risk/protective factors that contributed to the positive and negative associations found. In particular, future work should focus on specific types of pesticides to better characterize their potential association with AD. The results from this study advocate for the need to implement targeted public health surveillance, in particular for the aforementioned activities at risk (crop farming, viticulture, and fruit arboriculture).

### Supplementary Information

Below is the link to the electronic supplementary material.

Supplementary file1 (DOCX 509 KB)

Supplementary file2 (XLSX 38 KB)

## Data Availability

The data that supports the findings of this study is not publicly available. A reasonable request to the Mutualité Sociale Agricole (MSA) can be made, but restrictions apply to the availability of these data due to both the individual and medical nature of the data, which requires approval from both the MSA and the French independent administrative authority protecting privacy and personal data (CNIL). Further information is available from the corresponding author upon request.

## Abbreviations

95% CI: 95% Confidence interval
AD: Alzheimer’s disease
ADRD: Alzheimer’s disease and other related dementias
AGEIS: Autonomy, Gerontology, E-health, Imaging and Society (Autonomie, Gérontologie, E-santé, Imagerie et Société in French)
ANR: French National Research Agency (Agence Nationale de la Recherche in French)
ATC: Anatomical therapeutic chemical classification system
CNIL: Independent administrative authority protecting privacy and personal data
DDT: Dichlorodiphenyltrichloroethane
DEMENTIA: Detection and ExaMination of NEurodegeneraTIve diseases in Agriculture
FM: Farm manager
GP: General practitioner
HR: Hazard ratio
ICD-10: 10th revision of the International Statistical Classification of Diseases and Related Health Problems
IQR: Interquartile range
MA: Main analysis
MIAI: Multidisciplinary Institute in Artificial Intelligence
MSA: Mutualité Sociale Agricole (National Health Insurance Fund for Agricultural Workers and Farmers)
OP: Organophosphate
OR: Odds ratio
PD: Parkinson’s disease
RR: Relative risk
SA: Sensitivity analysis
SD: Arithmetic standard deviation
STROBE: Strengthening the Reporting of Observational Studies in Epidemiology
TRACTOR: Tracking and monitoring occupational risks in agriculture
UGA: Université Grenoble Alpes
UK: United Kingdom
US: United States of America
VIF: Variance inflation factor
