# Correction: Agricultural activities and risk of Alzheimer’s disease: the TRACTOR project, a nationwide retrospective cohort study

**DOI:** 10.1007/s10654-024-01116-6

**Published:** 2024-04-18

**Authors:** Pascal Petit, Elise Gondard, Gérald Gandon, Olivier Moreaud, Mathilde Sauvée, Vincent Bonneterre

**Affiliations:** 1 CNRS, UMR 5525, VetAgro Sup, Grenoble INP, CHU Grenoble Alpes, TIMC, Univ. Grenoble Alpes, Grenoble, 38000 France; 2 Present Address: Centre Regional de Pathologies, Professionnelles et Environnementales, CHU Grenoble Alpes, Grenoble, 38000 France; 3 Present Address: AGEIS, Univ. Grenoble Alpes, Grenoble, 38000 France; 4 Centre Mémoire de Ressources et de Recherche, CHU Grenoble Alpes, Grenoble, 38000 France; 5 Laboratoire de Psychologie et Neurocognition, UMR 5105, CNRS, LPNC, Univ. Grenoble Alpes, Univ. Savoie Mont Blanc, Grenoble, 38000 France

**Correction: European Journal of Epidemiology**

10.1007/s10654-023-01079-0

The article “Agricultural activities and risk of Alzheimer’s disease: the TRACTOR project, a nationwide retrospective cohort study”, written by Pascal Petit, Elise Gondard, Gérald Gandon, Olivier Moreaud, Mathilde Sauvée and Vincent Bonneterre, was originally published electronically on the publisher’s internet portal on 10 January 2024 without open access. With the author(s)’ decision to opt for Open Choice the copyright of the article changed on 08 March 2024 to © The Author(s) 2023 and the article is forthwith distributed under a Creative Commons Attribution 4.0 International License, which permits use, sharing, adaptation, distribution and reproduction in any medium or format, as long as you give appropriate credit to the original author(s) and the source, provide a link to the Creative Commons licence, and indicate if changes were made. The images or other third party material in this article are included in the article’s Creative Commons licence, unless indicated otherwise in a credit line to the material. If material is not included in the article’s Creative Commons licence and your intended use is not permitted by statutory regulation or exceeds the permitted use, you will need to obtain permission directly from the copyright holder. To view a copy of this licence, visit http://creativecommons.org/licenses/by/4.0.

The original article has been corrected.

