# Supplementary material for: Agricultural activities and risk of Alzheimer’s disease: the TRACTOR project, a nationwide retrospective cohort study
Source: Eur J Epidemiol. 2024 Jan 10;39(3):271–87. doi: 10.1007/s10654-023-01079-0 (PMC10995077; doi:10.1007/s10654-023-01079-0)
Supplement: Supplementary file 1 — Supplementary file1 (DOCX 509 KB) [file 10654_2023_1079_MOESM1_ESM.docx]

**Supplemental information**

**Title**

Agricultural activities and risk of Alzheimer’s disease: the TRACTOR project, a nationwide retrospective cohort study

**Authors**

Pascal Petit, Elise Gondard, Gérald Gandon, Olivier Moreaud, Mathilde Sauvée, Vincent Bonneterre

**Table of contents**

[Table 1 List and definition of agricultural activities available using MSA databases 2](#_Toc145927852)

[Table S2 List of potential covariates considered in the statistical analyses 3](#_Toc145927853)

[Table S3 Number of farm managers and identified cases for each analysis conducted 4](#_Toc145927854)

[Table S4 Number of farm managers with ICD-10 and ATC codes of interest 5](#_Toc145927855)

[Table S5 Agricultural activities and risks of AD, TRACTOR project, France, 2002-2016. Comparison of the main analysis with all sensitivity analyses for all sex categories. 6](#_Toc145927856)

[Figure S1 Agricultural activities and risks of AD among female farm managers, TRACTOR project, France, 2002-2016. Comparison of the main analysis with the sensitivity analyses. 7](#_Toc145927857)

[Figure S2 Agricultural activities and risks of AD among male farm managers, TRACTOR project, France, 2002-2016. Comparison of the main analysis with the sensitivity analyses. 9](#_Toc145927858)

[STROBE Statement Checklist 11](#_Toc145927859)

# **Table 1** List and definition of agricultural activities available using MSA databases

| **Activity name** | **Definition** | **Example of task** |
| --- | --- | --- |
| Truck farming, floriculture/flower-growing | Practice of growing and harvesting fruits, vegetables, and flowers for sale | Proper crop selection, soil and water management, pest and disease management, harvesting, and post-harvest handling |
| Fruit arboriculture | Cultivation of fruits growing on trees | Pruning branches, removing damaged or diseased branches, fertilizing trees, monitoring, and controlling pests and diseases |
| Garden center/tree nursery | Propagation, breeding and growing of plants and trees to a desired size | Planting, watering, weeding, digging, and using tools such as a sieve, or spade |
| Crop farming (e.g. wheat, corn, and industrial grower) | Cultivation of plants (e.g. cereals, vegetables) for food | Monitoring and controlling pests and diseases, fertilizing, harvesting and post-harvest handling, grading, soil and water management, tractor driving |
| Viticulture | Cultivation and harvesting of grapes, and winemaking | Monitoring and controlling pests and diseases, fertilizing, soil and water management, canopy management, monitoring fruit development and characteristics, harvesting and post-harvest handling, and vine pruning |
| Sylviculture/forestry (e.g. thinning, pruning) | Controlling the growth, composition/structure, as well as quality of forests | Thinning, pruning, and spacing |
| Unspecified specialized farming (e.g. herbs, mushrooms) | Cultivation and harvesting of herbs, spices, medical plants, and mushrooms | Layering, cutting, grafting, pruning, mulching, and weed management |
| Dairy farming | Raising and breeding of domestic cows for milk production | Breeding, milking (manually, or using a machine), feeding, bulk handling, disease prevention, and managing waste |
| Cow farming | Raising and breeding of domestic cows as livestock | Feeding, maintaining facilities, monitoring the herd for signs of illness, assisting with calving, performing artificial insemination, and managing waste |
| Both/mixed dairy and cow farming | Raising and breeding of domestic cows as livestock and for milk production | Breeding, milking, feeding, bulk handling, and disease prevention |
| Ovine and caprine farming | Raising and breeding of domestic sheep and goats | Breeding, milking, wool production, maintenance of the property, mustering, milking, shearing and drenching livestock, sterilizing machines, and collecting, grading and packaging produce |
| Pig farming | Raising and breeding of domestic pigs as livestock | Maintain equipment and buildings, spread manure, feeding, monitoring the pigs for signs of illness, assist in artificial insemination, and transporting stock to farms or processing plants |
| Stud farming | Raising and breeding of domestic horses | Feeding, breeding, assist in artificial insemination, and disease prevention |
| Unspecified large animal farming (e.g. ostrich, llama) | Raising and breeding of large animals such as wild animals, ostrich, bison, boar, or llama | Feeding, breeding, and disease prevention |
| Poultry and rabbit farming | Raising and breeding of domestic birds and rabbits | Feeding, breeding, disease prevention, assist in artificial insemination, gathering eggs, cleaning rooms and equipment/facility maintenance |
| Unspecified small animal farming (e.g. frogs, snails, bees) | Raising and breeding of small animals such as frogs, bees, insects, minks, beavers, or snails | Feeding, breeding, and disease prevention |
| Training, dressage, riding clubs | Activity including training and dressage of domestic equines | Feeding, disease prevention, teaching, maintenance of the property, and tourism |
| Shellfish farming (e.g. oyster farming, scallop aquaculture) | Cultivation and harvest of aquatic invertebrates, such as oysters, clams, and mussels | Breeding, rearing, harvesting, using a rack, processing, and shipment |
| Unspecified and mixed farming (e.g. polyculture, mixed farming, diversified farming) | Activity involving both the growing of crops and the raising of livestock, or the growing of more than one crop species | Planning and coordinating the production and marketing of crops and livestock, breeding and raising livestock for the production of meat, wool, skins, milk or eggs, and growing grain, seed crops, vegetables, fruit or nuts |
| Salt works/salt evaporation pond | Production of salt from sea water | Harvesting, and filtering |
| Wood production (e.g. lopping) | Harvesting of round wood (trunk and branch wood) from coniferous (softwood) and non-coniferous (hardwood) trees | Harvesting, lopping, and transport |
| Stationary sawmill (e.g. edging, trimming, decking, debarking) | Cutting logs into lumber using large bandsaw blades | Edging, trimming, decking, debarking, use of machines |
| Agricultural work companies (e.g. pesticide applications, harvest reaping) | Companies that assist or perform farm works such as harvest reaping | Harvest reaping, harrowing, sowing seeds, pesticide application, tilling, and clearing |
| Gardening, landscaping and reforestation companies | Companies that perform gardening, landscaping and reforestation work | Gardening, landscaping, chainsaw and skidder operation, climbing on trees, monitoring and controlling pests and diseases |
| Company representative/authorized representative | Self-employed insurance broker responsible for representing a legal entity (agricultural insurance company) | Traveling, administrative tasks, and visiting farms |
| Rural craftsperson (e.g. mason, mechanics) | Self-employed person performing construction or hand-crafted work in rural areas, including jobs such as well-diggers, painters, electricians, masons, carpenters, saddlers, blacksmiths, mechanics, wheelwright, or farriers | Fixing agricultural machinery, painting, welding, and hammering |

# **Table S2** List of potential covariates considered in the statistical analyses

| **Dependent variable** | **Modality** |
| --- | --- |
| AD diagnosis (LTI declaration or drug prescription) | 2 categories: yes or no |
| Time to first AD insurance declaration, or drug prescription | continuous |
|  |  |
| **Independent variables – covariates always included** |  |
| Activity | 2 categories: yes or no |
| Sex^*^ | 2 categories: female or male |
| Age | continuous |
|  |  |
| **Independent variables – potential covariates selected based on VIF** |  |
| First year of the farm’s establishment, years^†^ | 4 categories:  before 1985;  1985-1994;  1995-2004;  after 2004 |
| Median yearly farm surface, hectares | 5 categories:  Farm surface = 0 hectares;  0 < farm surface < 5 hectares;  5 ≤ farm surface < 25 hectares;  25 ≤ farm surface < 50 hectares;  Farm surface ≥ 50 hectares |
| Median yearly insurance premium, euros | 5 categories:  insurance premiums = 0 euro;  0 < insurance premiums <1500 euros;  1500 ≤ insurance premiums < 5000 euros;  5000 ≤ insurance premiums < 10000 euros;  Insurance premiums ≥ 10000 euros |
| Number of associates**^‡^** | 3 categories: 0, 1, > 1 |
| Unemployment status | 2 categories: never unemployed or had been unemployed at least once over the period 2002-2016 |
| Number of farms | 2 categories: 1 farm or > 1 farm |
| Family status | 2 categories: single or as a couple |
| Partner work status | 2 categories: perform or do not perform task to help farm manager |
| Having a secondary activity**^‡^** | 2 categories: yes or no |
| Number of pre-existing medical comorbidities | 3 categories:  0 comorbidity;  1 comorbidity;  > 1 comorbidity |
| Farm location | 13 categories: 13 metropolitan French administrative geographical areas |

^*^The analysis was adjusted on sex only for “both sexes”; otherwise, the sex was used for subgroup analyses.

^†^ Main analysis: this variable was always included in the models for sensitivity analysis 1 (SA1).

**^‡^** A FM can have associate(s) in the case of farm clustering (the grouping of farms belonging to several FMs), or in the case of a farm owned by several persons. A secondary activity is defined as an agricultural activity (e.g. grassland farming) that a FM can perform in addition to its main activity (e.g. ovine farming). The nature of the secondary activity is, however, unknown.

# **Table S3** Number of farm managers and identified cases for each analysis conducted

| **Analysis** | **FMs without AD** No. (%) | **FMs with AD** No. (%) | **Total number of FMs** |
| --- | --- | --- | --- |
| Main analysis | 1031002 (99.5%) | 5067 (0.5%) | 1036069 |
| Sensitivity analysis 1 - using the same covariates for all models | 1031002 (99.5%) | 5067 (0.5%) | 1036069 |
| Sensitivity analysis 2 - only including individual ≥ 60 years | 156714 (97.4%) | 4229 (2.6%) | 160943 |
| Sensitivity analysis 3 - Alzheimer's disease and related dementias | 1030639 (99.5%) | 5430 (0.5%) | 1036069 |
| Sensitivity analysis 4 - not including individuals with LTI for PD | 1031263 (99.5%) | 4806 (0.5%) | 1036069 |
| Sensitivity analysis 5 - AD without individual with PD, or F03 | 1032183 (99.6%) | 3886 (0.4%) | 1036069 |
| Sensitivity analysis 6 - AD identification only using LTI declaration data | 1031929 (99.6%) | 4140 (0.4%) | 1036069 |
| Sensitivity analysis 7 - AD identification only using drug prescription data | 1033460 (99.7%) | 2609 (0.3%) | 1036069 |

Abbreviations: AD: Alzheimer’s disease, FM: farm manager, No: number of, PD: Parkinson’s disease.

# **Table S4** Number of farm managers with ICD-10 and ATC codes of interest

A- Farm managers with a LTI declaration for an ICD-10 code of interest

| **ICD-10 code** | **Definition** | **Number of farm managers**  No. (%) |
| --- | --- | --- |
| F00, F01, F02, F03 and/or G30 | Alzheimer’s disease and other related dementias (ADRDs) | 4538 (83.6) |
| F00, F03 and/or G30 | Alzheimer’s disease | 4171 (82.3) |
| F00 and/or G30 | Alzheimer’s disease | 2758 (54.4) |
| F00 | Dementia in Alzheimer’s disease | 2598 (51.3) |
| F03 | Unspecified dementia | 1471 (29.0) |
| F01 | Vascular dementia | 384 (7.6) |
| G30 | Alzheimer’s disease | 198 (3.9) |
| F02 | Dementia in other diseases classified elsewhere | 10 (0.2) |

B - Farm managers with a drug prescription for an ATC code of interest

| **ATC code** | **Number of FMs without**  **a LTI**  No. (%) | **Number of FMs with a LTI declaration for:** | | | | | | | | | | | | |
| --- | --- | --- | --- | --- | --- | --- | --- | --- | --- | --- | --- | --- | --- | --- |
|  |  | **F00**  No. (%) | **F01**  No. (%) | **F02**  No. (%) | **F03**  No. (%) | **G20**  No. (%) | **G21**  No. (%) | **G22**  No. (%) | **G20, G21**  **and/or G22**  No. (%) | **G30**  No. (%) | **F00 and/or**  **G30**  No. (%) | **F00, F03**  **and/or G30**  No. (%) | **(F00, F03 or G30)**  **and (G20 or G21)**  No. (%) | **F00, F01, F02,**  **F03 and/or G30**  No. (%) |
| N06DX01 (memantine) - overall^*^ | 260 (5.1) | 715 (14.1) | 55 (1.1) | 2 (0.04) | 197 (3.9) | 36 (0.7) | 2 (0.04) | 0 | 38 (0.8) | 75 (1.48) | 773 (15.3) | 954 (18.8) | 18 (0.4) | 1002 (18.5) |
| N06DA03 (rivastigmine) - overall^*^ | 152 (3.0) | 563 (11.1) | 37 (0.7) | 2 (0.04) | 184 (3.6) | 174 (3.4) | 4 (0.08) | 0 | 178 (3.5) | 45 (0.9) | 595 (11.7) | 770 (15.2) | 56 (1.1) | 802 (14.8) |
| N06DA02 (donepezil) - overall^*^ | 130 (2.6) | 416 (8.2) | 23 (0.5) | 1 (0.02) | 74 (1.5) | 18 (0.4) | 0 | 0 | 18 (0.4) | 46 (0.9) | 453 (8.9) | 515 (10.2) | 3 (0.06) | 536 (9.9) |
| N06DA04 (galantamine) - overall^*^ | 35 (0.7) | 172 (3.4) | 10 (0.2) | 1 (0.02) | 31 (0.6) | 5 (0.1) | 0 | 0 | 5 (0.1) | 33 (0.7) | 198 (3.9) | 226 (4.5) | 2 (0.04) | 234 (4.3) |
| N06DA52 - overall^*^ | 0 | 0 | 0 | 0 | 0 | 0 | 0 | 0 | 0 | 0 | 0 | 0 | 0 | 0 |
| N06DA53 - overall^*^ | 0 | 0 | 0 | 0 | 0 | 0 | 0 | 0 | 0 | 0 | 0 | 0 | 0 | 0 |
| N06DX01, N06DA03, N06DA03 or N06DA04 | 501 (9.9) | 1480 (29.2) | 106 (2.1) | 4 (0.08) | 422 (8.3) | 212 (4.2) | 6 (0.1) | 0 | 218 (4.3) | 148 (2.9) | 1594 (31.5) | 1984 (39.2) | 71 (1.4) | 2075 (38.2) |
| N06DX01 (memantine) alone^†^ | 190 (3.7) | 398 (7.9) | 38 (0.8) | 1 (0.02) | 145 (2.9) | 19 (0.4) | 2 (0.04) | 0 | 21 (0.4) | 30 (0.6) | 422 (8.3) | 558 (11.0) | 10 (0.2) | 591 (10.9) |
| N06DA02 (donepezil) alone^†^ | 99 (2.0) | 237 (4.7) | 16 (0.3) | 0 | 49 (1.0) | 11 (0.2) | 0 | 0 | 11 (0.2) | 30 (0.6) | 261 (5.2) | 302 (6.0) | 0 | 316 (5.8) |
| N06DA03 (rivastigmine) alone^†^ | 109 (2.2) | 369 (7.3) | 27 (0.5) | 2 (0.04) | 147 (2.9) | 156 (3.1) | 4 (0.08) | 0 | 160 (3.2) | 22 (0.4) | 386 (7.6) | 527 (10.4) | 50 (1.0) | 550 (10.1) |
| N06DA04 (galantamine) alone^†^ | 27 (0.5) | 115 (2.3) | 6 (0.1) | 0 | 22 (0.4) | 5 (0.1) | 0 | 0 | 5 (0.1) | 19 (0.4) | 128 (2.5) | 149 (2.9) | 3 (0.06) | 153 (2.8) |
| N06DX01+N06DA02 | 26 (0.5) | 124 (2.5) | 6 (0.1) | 0 | 16 (0.3) | 3 (0.06) | 0 | 0 | 3 (0.06) | 13 (0.3) | 135 (2.7) | 147 (2.9) | 2 (0.04) | 152 (2.8) |
| N06DA03+N06DX01 | 37 (0.7) | 133 (2.6) | 8 (0.2) | 0 | 27 (0.5) | 14 (0.3) | 0 | 0 | 14 (0.3) | 18 (0.4) | 144 (2.8) | 169 (3.3) | 6 (0.1) | 176 (3.2) |
| N06DX01+N06DA04 | 7 (0.1) | 36 (0.7) | 3 (0.06) | 0 | 5 (0.1) | 0 | 0 | 0 | 0 | 11 (0.2) | 46 (0.9) | 50 (1.0) | 0 | 52 (1.0) |
| N06DA02+N06DA03 | 5 (0.1) | 30 (0.6) | 1 (0.02) | 0 | 4 (0.08) | 4 (0.08) | 0 | 0 | 4 (0.08) | 0 | 30 (0.6) | 34 (0.7) | 0 | 35 (0.6) |
| N06DA02+N06DA04 | 0 | 4 (0.08) | 0 | 0 | 1 (0.02) | 0 | 0 | 0 | 0 | 0 | 4 (0.08) | 5 (0.1) | 0 | 5 (0.09) |
| N06DA03+N06DA04 | 1 (0.02) | 9 (0.2) | 1 (0.02) | 0 | 2 (0.04) | 0 | 0 | 0 | 0 | 2 (0.04) | 11 (0.2) | 12 (0.2) | 0 | 13 (0.2) |
| N06DA03+N06DA04+N06DX01 | 1 (0.02) | 3 (0.06) | 0 | 0 | 0 | 0 | 0 | 0 | 0 | 0 | 3 (0.06) | 3 (0.06) | 0 | 3 (0.06) |
| N06DA02+N06DA04+N06DX01 | 1 (0.02) | 3 (0.06) | 0 | 1 (0.02) | 0 | 0 | 0 | 0 | 0 | 0 | 3 (0.06) | 3 (0.06) | 0 | 4 (0.07) |
| N06DA02+N06DA03+N06DX01 | 2 (0.04) | 18 (0.4) | 0 | 0 | 3 (0.06) | 0 | 0 | 0 | 0 | 2 (0.04) | 19 (0.4) | 22 (0.4) | 0 | 22 (0.4) |
| N06DA02+N06DA03+N06DA04 | 1 (0.02) | 1 (0.02) | 0 | 0 | 0 | 0 | 0 | 0 | 0 | 0 | 1 (0.02) | 1 (0.02) | 0 | 1 (0.04) |
| N06DX01+N06DA03+N06DA03+N06DA04 | 0 | 0 | 0 | 0 | 1 (0.02) | 0 | 0 | 0 | 0 | 1 (0.02) | 1 (0.02) | 2 (0.04) | 0 | 2 (0.02) |

Abbreviations: AD: Alzheimer’s disease, ATC: Anatomical Therapeutic Chemical classification system, F00: dementia in Alzheimer’s disease, F01: vascular dementia, F02: dementia in other diseases classified elsewhere, F03: unspecified dementia, FM: farm managers, G20: Parkinson's disease, G21: secondary parkinsonism, G22: Parkinsonism in diseases classified elsewhere, G30: Alzheimer’s disease, LTI: long-term illness declaration, ICD: 10^th^ revision of the International Statistical Classification of Diseases and Related Health Problems, N06DX01: memantine, N06DA02: donepezil, N06DA03: rivastigmine, N06DA04: galantamine, N06DA52: donepezil and memantine, N06DA53: donepezil, memantine and ginkgo folium.

^*^Farm managers that were prescribed with a given drug at least once. ^†^ Farm managers that were prescribed with only one type of drugs (the drug specified).

# **Table S5** Agricultural activities and risks of AD, TRACTOR project, France, 2002-2016. Comparison of the main analysis with all sensitivity analyses for all sex categories.

Please refer to the MS Excel file entitled 10654_2023_1079_MOESM2_ESM.


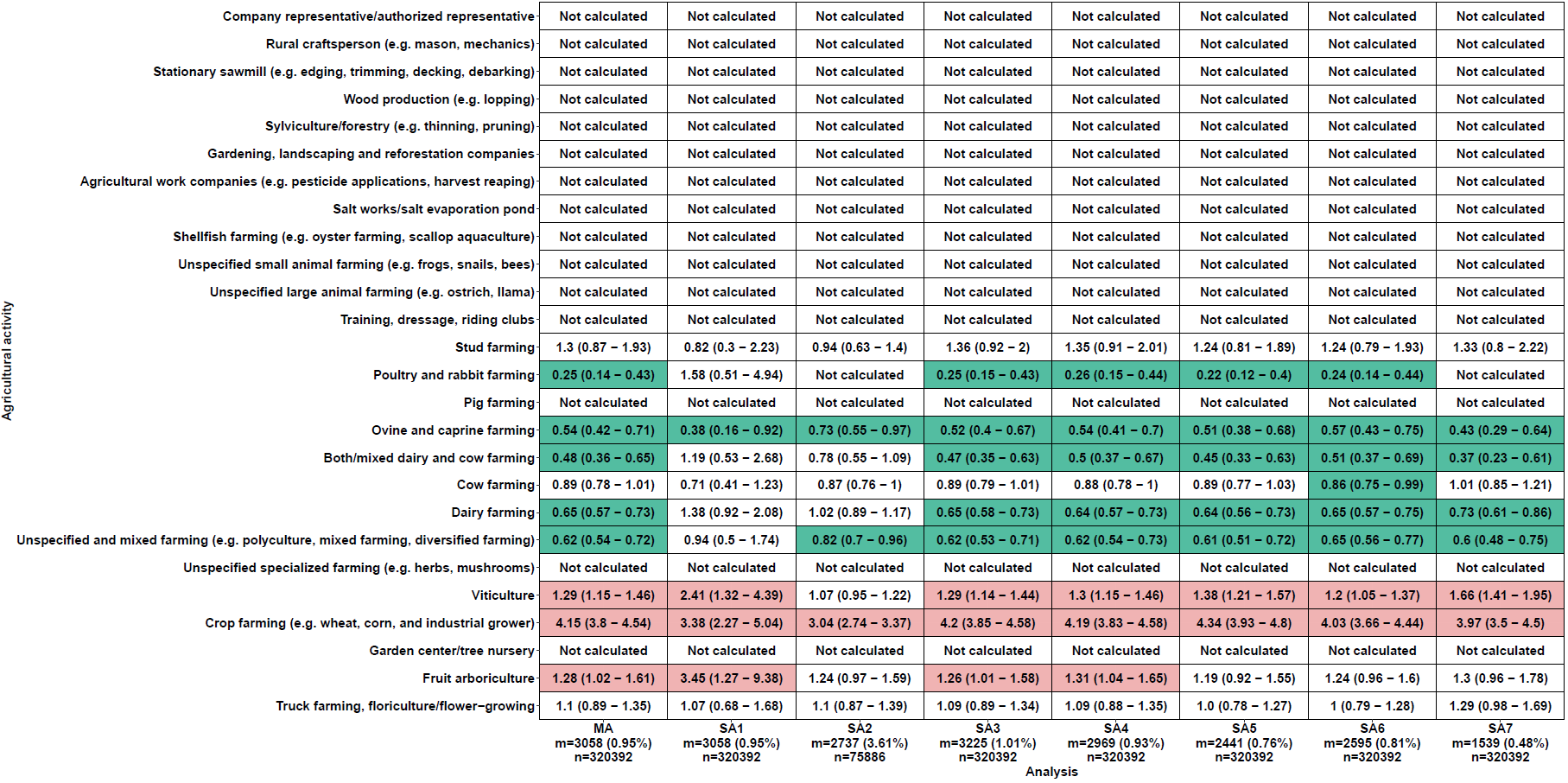


# **Figure S1** Agricultural activities and risks of AD among female farm managers, TRACTOR project, France, 2002-2016. Comparison of the main analysis with the sensitivity analyses.

Multivariable Cox regression models for Alzheimer’s disease according to different analyses (x-axis) for which each agricultural activity (y-axis) are displayed when the number of exposed cases was sufficient (m ≥ 10). The hazard ratio is provided with a 95% confidence interval for each analysis. The red cells refer to a higher risk of Alzheimer’s disease, while the green cells represent a lower risk of Alzheimer’s disease. The white cells indicate situations where there is no difference in risk of Alzheimer’s disease among the farm managers performing the considered activity compared to the population of farm managers not performing the considered activity.

Abbreviations: m: total number of cases, MA: main analysis, n: total number of FMs, SA: sensitivity analysis.

MA: adjusted for sex (for “both sexes” only), age, first year of the farm’s establishment, farm surface, earnings, number of associates, unemployment status, total number of farms, family status, partner work status, farm location, number of comorbidities, and having a secondary activity.

SA1: adjusted for sex (for “both sexes” only), age, and the first year of the farm’s establishment for all models.

SA2: included only FMs who were 60 years and older, and adjusted for the same variable as the main analysis.

SA3: included FMs diagnosed with AD and other related dementias, and adjusted for the same variable as the main analysis.

SA4: adjusted for the same variable as the main analysis, but FMs who were prescribed with an AD drug that also had a LTI declaration for PD were not considered as AD cases.

SA5: adjusted for the same variable as the main analysis, but FMs who were prescribed with an AD drug that also had a LTI declaration for PD or a LTI for unspecified dementia were not considered as AD cases.

SA6: adjusted for the same variable as the main analysis, but the AD case identification was restricted to LTI declarations.

SA7: adjusted for the same variable as the main analysis, but the AD case identification was restricted to drug prescriptions.


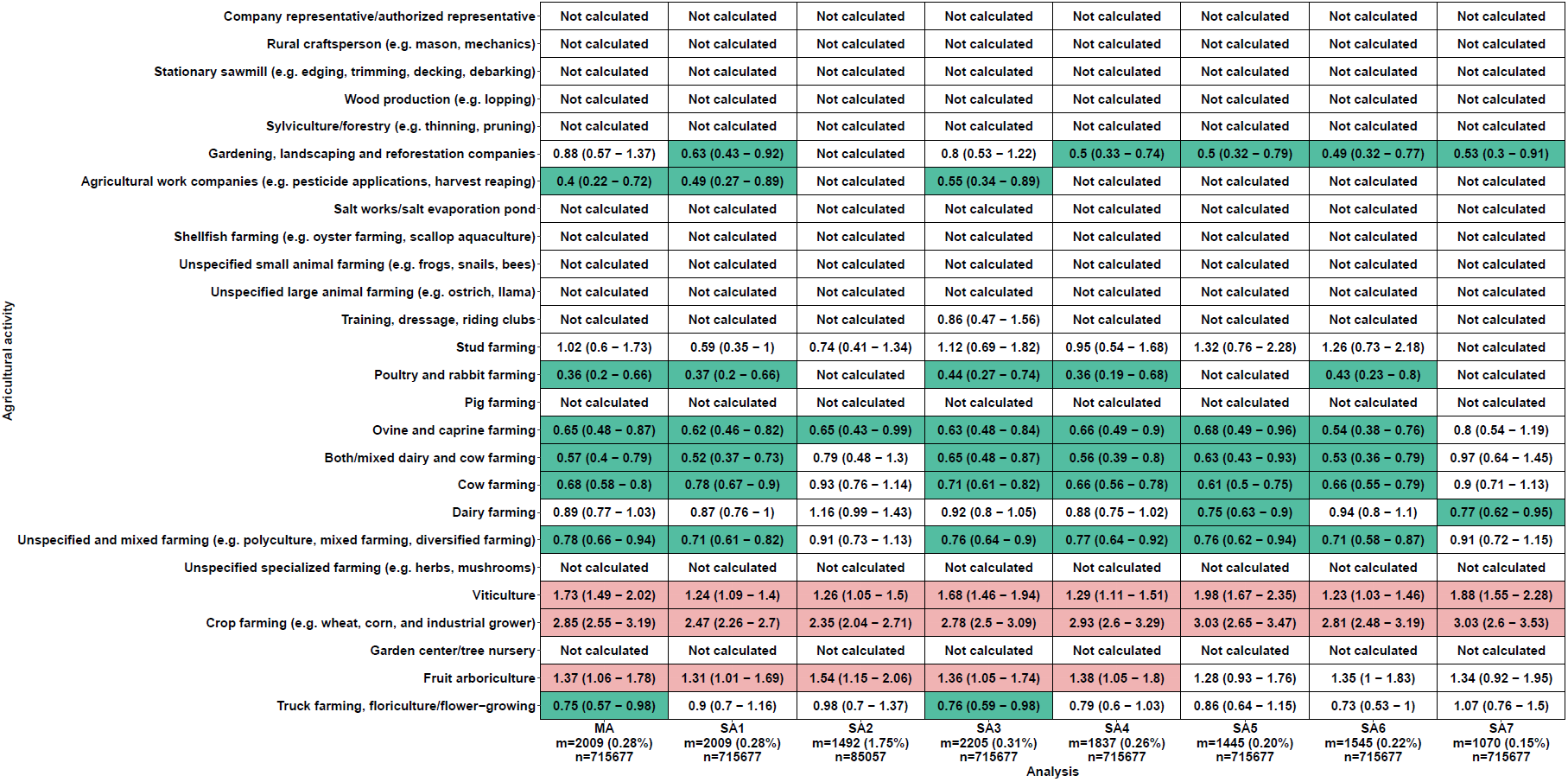


# **Figure S2** Agricultural activities and risks of AD among male farm managers, TRACTOR project, France, 2002-2016. Comparison of the main analysis with the sensitivity analyses.

Multivariable Cox regression models for Alzheimer’s disease according to different analyses (x-axis) for which each agricultural activity (y-axis) are displayed when the number of exposed cases was sufficient (m ≥ 10). The hazard ratio is provided with a 95% confidence interval for each analysis. The red cells refer to a higher risk of Alzheimer’s disease, while the green cells represent a lower risk of Alzheimer’s disease. The white cells indicate situations where there is no difference in risk of Alzheimer’s disease among the farm managers performing the considered activity compared to the population of farm managers not performing the considered activity.

Abbreviations: m: total number of cases, MA: main analysis, n: total number of FMs, SA: sensitivity analysis.

MA: adjusted for sex (for “both sexes” only), age, first year of the farm’s establishment, farm surface, earnings, number of associates, unemployment status, total number of farms, family status, partner work status, farm location, number of comorbidities, and having a secondary activity.

SA1: adjusted for sex (for “both sexes” only), age, and the first year of the farm’s establishment for all models.

SA2: included only FMs who were 60 years and older, and adjusted for the same variable as the main analysis.

SA3: included FMs diagnosed with AD and other related dementias, and adjusted for the same variable as the main analysis.

SA4: adjusted for the same variable as the main analysis, but FMs who were prescribed with an AD drug that also had a LTI declaration for PD were not considered as AD cases.

SA5: adjusted for the same variable as the main analysis, but FMs who were prescribed with an AD drug that also had a LTI declaration for PD or a LTI for unspecified dementia were not considered as AD cases.

SA6: adjusted for the same variable as the main analysis, but the AD case identification was restricted to LTI declarations.

SA7: adjusted for the same variable as the main analysis, but the AD case identification was restricted to drug prescriptions.

# **STROBE Statement Checklist**

|  | Item  No | Recommendation | Page |
| --- | --- | --- | --- |
| **Title and abstract** | 1 | (*a*) Indicate the study’s design with a commonly used term in the title or the abstract | 1, 4 |
|  |  | (*b*) Provide in the abstract an informative and balanced summary of what was done and what was found | 4 |
| Introduction | | |  |
| Background/rationale | 2 | Explain the scientific background and rationale for the investigation being reported | 7 |
| Objectives | 3 | State specific objectives, including any prespecified hypotheses | 7, 8 |
| Methods | | |  |
| Study design | 4 | Present key elements of study design early in the paper | 8, 9 |
| Setting | 5 | Describe the setting, locations, and relevant dates, including periods of recruitment, exposure, follow-up, and data collection | 8, 9 |
| Participants | 6 | (*a*) Give the eligibility criteria, and the sources and methods of selection of participants. Describe methods of follow-up | 9 |
|  |  | (*b*) For matched studies, give matching criteria and number of exposed and unexposed | DNA |
| Variables | 7 | Clearly define all outcomes, exposures, predictors, potential confounders, and effect modifiers. Give diagnostic criteria, if applicable | 8-11 |
| Data sources/ measurement | 8* | For each variable of interest, give sources of data and details of methods of assessment (measurement). Describe comparability of assessment methods if there is more than one group | 8-11 |
| Bias | 9 | Describe any efforts to address potential sources of bias | 10, 11 |
| Study size | 10 | Explain how the study size was arrived at | 8-11 |
| Quantitative variables | 11 | Explain how quantitative variables were handled in the analyses. If applicable, describe which groupings were chosen and why | 8-11, suppl |
| Statistical methods | 12 | (*a*) Describe all statistical methods, including those used to control for confounding | 10, 11 |
|  |  | (*b*) Describe any methods used to examine subgroups and interactions | 10, 11 |
|  |  | (*c*) Explain how missing data were addressed | 11 |
|  |  | (*d*) If applicable, explain how loss to follow-up was addressed | DNA |
|  |  | (*e*) Describe any sensitivity analyses | 11 |
| Results | | |  |
| Participants | 13* | (a) Report numbers of individuals at each stage of study-e.g., numbers potentially eligible, examined for eligibility, confirmed eligible, included in the study, and analyzed | 11-13 |
|  |  | (b) Give reasons for non-participation at each stage | DNA |
|  |  | (c) Consider use of a flow diagram | DNA |
| Descriptive data | 14* | (a) Give characteristics of study participants (e.g., demographic, clinical, social) and information on exposures and potential confounders | 11-13, Table 2 |
|  |  | (b) Indicate number of participants with missing data for each variable of interest | DNA |
|  |  | (c) Summarize follow-up time (e.g., average and total amount) | 12 |
| Outcome data | 15* | Report numbers of outcome events or summary measures over time | 14-20, suppl |
| Main results | 16 | (*a*) Give unadjusted estimates and, if applicable, confounder-adjusted estimates and their precision (e.g., 95% confidence interval). Make clear which confounders were adjusted for and why they were included | 14-20, suppl |
|  |  | (*b*) Report category boundaries when continuous variables were categorized | DNA |
|  |  | (*c*) If relevant, consider translating estimates of relative risk into absolute risk for a meaningful time period | DNA |
| Other analyses | 17 | Report other analyses done-e.g., analyses of subgroups and interactions, and sensitivity analyses | 14-20, suppl |
| Discussion | | |  |
| Key results | 18 | Summarize key results with reference to study objectives | 21 |
| Limitations | 19 | Discuss limitations of the study, taking into account sources of potential bias or imprecision. Discuss both direction and magnitude of any potential bias | 25-27 |
| Interpretation | 20 | Give a cautious overall interpretation of results considering objectives, limitations, multiplicity of analyses, results from similar studies, and other relevant evidence | 22-27 |
| Generalizability | 21 | Discuss the generalizability (external validity) of the study results | 27 |
| Other information | | |  |
| Funding | 22 | Give the source of funding and the role of the funders for the present study and, if applicable, for the original study on which the present article is based | 3 |

*Give information separately for exposed and unexposed groups.

DNA: does not applied, suppl: supplementary information.
